# Stories of Bereavement: Examining Medical Students’ Reflections on Loss and Grief

**DOI:** 10.1177/00302228241305829

**Published:** 2024-12-01

**Authors:** Johanna Shapiro, Nicholas Freeman, Alexis Nguyen, Nancy Dang, Yasaman Lorkalantari

**Affiliations:** Department of Family Medicine, School of Medicine, University of California, Irvine, CA, USA

**Keywords:** medical students, loss, grief, medical education, qualitative research

## Abstract

In this qualitative study, we used thematic analysis to examine reflective essay data from students at a United States public medical school (*N* = 60). We sought to identify how students describe their experiences of loss and grief, as well as examine differences between students’ responses to personal and professional loss. Students in both groups reported similar reactions with some expected differences: Students who experienced professional loss more often emphasized skills focused on helping patients’ families. In accounts of personal loss, students appeared more focused on their own grief and helplessness. Students in both groups noted many positive as well as negative reactions. Students recounting both types of loss reported little about how they coped with grief or about receiving either informal or institutional support. Medical educators should help students develop effective coping skills in response to loss and provide better institutional support during times of bereavement.

## Introduction

Overwhelming experiences, such as loss through the death of a patient or family member, can have many deleterious effects on medical students, as well as some positive learning outcomes (Price et al., 2022). Although research on implications of medical students’ personal loss is scant, studies suggest both negative consequences on learning and patient care, but also possible benefits including more realistic and emotionally centered attitudes toward end of life (EOL) care (Whyte et al., 2013).

The literature regarding medical students’ experiences of professional loss is more robust and documents many negative emotional responses. One study identified emotional reactions of hurt, anger, frustration, remorse, sadness, guilt, unhappiness, hopelessness, helplessness and powerlessness. At times students felt responsible or guilty for not succeeding in saving patients’ lives (Maisa et al., 2010). Other studies identified similar negative reactions. A study comparing Inpatient and Emergency Department (ED) student reactions to loss concluded that Inpatient experiences resulted in sadness, helplessness, grief, disappointment, irritation, feeling overwhelmed, traumatized, upset, anxious, and heartbroken; the ED experience led to feelings of frustration, shock, powerlessness, confusion, surprise, and numbness (Batley et al., 2017). Other loss emotions reported include disgust, guilt, sadness, shock, and indifference (Kelly & Nisker, 2010). Complicated grief in medical students, including excessive focus on restoring normalcy combined with difficulty sleeping, depression, anxiety and disruption in interpersonal relationships, resulted in distancing from dying patients (Ferguson et al., 2023).

Students were concerned with knowing how to manage their strong emotion as well as those of patients and families in response to death and dying They were conflicted about whether they should “feel something” or simply carry on with their duties. They also worried about upsetting patients/families by “saying the wrong thing” (Gillett et al., 2016). The stress of encountering death at times could result in students losing patient-centeredness (Bombeke et al., 2010).

A few studies reported more mixed results. One found that while some students felt confused and distressed, others developed greater appreciation for life, strengthened their religious beliefs, felt more comfortable with death, found their emotions to be manageable, and were more accepting of their abilities as medical professionals. While some felt desensitized and detached, others began to view patients more holistically, some felt unsure about their role and had a hard time empathizing, but others felt a patient’s death allowed them to improve their communication, patience, and compassion (Ho et al., 2020). A different study concluded that a small majority of medical students interviewed did not want to work with dying patients because they wished to avoid negative emotions and felt they did not have either the communication or coping skills necessary to care for this population; but the remaining students were positively motivated to work with patients at end of life (Dobrowolska et al., 2019).

Student coping mechanisms in response to loss included religion, taking time off, denial, solation, and submerging themselves in standard medical behaviors (Ho et al., 2020) as well as staying busy, processing negative feelings, normalization, and discussing thoughts and feelings with others (Trivate et al., 2019). Sometimes students tried to avoid all grief reactions in response to patient death (Maisa et al., 2010) and relied primarily on coping strategies of distancing, detachment and being involved in other things (Batley et al., 2017). It seemed that an ‘unexpected’ death often could be resolved for students by turning the event into a learning experience, which the study authors speculate helped to offset loss of control (Kelly & Nisker, 2010).

In terms of institutional support, the Ho et al. study (2020) found that some students felt they were not adequately supported and that their medical team was insensitive, whereas others felt it beneficial to talk with their seniors and saw other professionals as role models. Other studies found that both negative and positive attending/resident role modeling was important in influencing students’ attitudes and behaviors toward end of life situations (Ratanawongsa et al., 2005); and that students felt supervisor support could improve subsequent professional practice (Nantha Kumar & Nyatsuro, 2020).

Regarding curricular preparation, students reported that lectures presenting theories of death and dying and skills of EOL communication did not always adequately prepare them for real end of life care. Students stressed the importance of learning through experience but felt there were few structured opportunities for discussing EOL situations they encountered (Gillett et al., 2016). Appropriate instructional activities, such as small group modules involving self-reflection, simulation, role-play and discussion of students’ experiences resulted in decreased negative emotional consequences (Sikstrom et al., 2019).

We considered common theoretical models of loss and grief to guide development of the codebook and coding. Kubler-Ross’ classic model introduced the five stages of grief: shock and denial; anger, resentment and guilt; bargaining; depression; and acceptance (Corr, 2021). These stages were often interpreted as distinct and sequential. As a result, the model has been empirically rejected for its failure to account for the complexity and diversity of the grieving process (Corr, 2021; Stroebe et al., 2017).

Worden’s model (2018) proposed an active process through four tasks of mourning: accepting the reality of the loss; processing the pain of grief; adjusting to a world without the deceased; and finding a connection with the deceased. These four tasks may be independently accomplished, fluctuate, and interact with each other. They may be completed in any order and the bereaved may return to them in the future (Rothaupt & Becker, 2007). Worden (2018) also suggested contextual factors to consider in better understanding the grief experience.

Stroebe and Schut’s (2010) Dual Process model suggested an oscillation between loss orientation and restoration orientation. This oscillation refers to the regulation of emotions between grieving loss and functioning in daily life. Loss orientation is emotion-focused coping in which the bereaved directly acknowledges the loss and experiences pain. In loss orientation, negative affect is predominant in early bereavement while positive affect serves an important role during the recovery from loss (Schut, 1999). Restoration orientation is problem-focused, managing secondary stressors resulting from the loss and rebuilding everyday life.

The goals of this research were as follows: (1) To identify how medical students described their experiences of loss and grief in terms of emotional responses, coping, and lessons learned. While many models of loss and grief exist, we still lack a conceptual model derived from medical students’ own observations and accounts. (2) To determine what differences between students’ responses to personal loss and professional loss could be identified. To our knowledge, no study has directly compared differences and similarities in medical students’ experiences of personal and professional loss.

## Methods

### Study Setting and Data Collection

This study drew on reflective essay data from students at a U.S. public medical school, enrolled in a fourth-year Art of Doctoring elective course. The essays were required and reviewed by faculty but did not influence the students’ grades. Students wrote short essays about personal experiences of loss and grief, either as a result of a patient death (professional) or the death of a family member or friend (personal), in response to this prompt:

“Write a couple of paragraphs on a personal or professional experience of loss and grief. Elaborate on how this experience informed your approach to patient care.”

### Recruitment

Students who matriculated through the elective received an email asking permission for the anonymous use of their essays in a research study about loss and grief, which was described in detail. They could decline participation by email. From the collection of approved assignments, we randomly selected 30 professional loss and 30 personal loss essays across six different fourth-year classes.

While in qualitative research there are not clear-cut guidelines to determine sample size similar to those derived from statistics-based rules in quantitative studies, we followed general recommendations from experienced qualitative researchers (Vasileiou et al., 2018). For example, more complex or multi-site studies often benefit from larger samples (Hagaman & Wutich, 2017). We were guided by the concept of theoretical saturation, the most widely used method of determining sample size in qualitative research, which emphasizes continuing data analysis until the point at which no new theoretical insights are emerging.

Because we had limited access to our cases (i.e., only the written essays) and thus could not reinterview or develop additional question probes, we opted for a somewhat larger sample size of 60 students to ensure that we had in fact achieved theoretical saturation. Further, because we were comparing two groups (personal loss and professional loss) that we hypothesized might be quite different on some dimensions, we chose to consider these two groups separately for analytic purposes, leading to a total sample of 60. We also wanted to be sure that we achieved not only coding saturation, but *meaning* saturation, which generally requires a larger sample (Henninck et al., 2017).

### Data Analysis

Using the qualitative data analysis software, Dedoose, we engaged in flexible coding (Deterding & Waters, 2021) to inductively code essays, where codes were developed from existing literature, researcher insights, and the essays themselves. Thematic analysis of essays was selected because reflective writing provides insights into students’ perceptions of challenging clinical encounters (Wald et al., 2019) and the process of professional identity formation (Hatem & Halpin, 2019). Over a six-month period, we analyzed 60 essays until achieving theoretical saturation of the data (i.e., no new themes were identified).

We coded for students’ emotional reactions, coping strategies and lessons learned. Emotional reactions encompassed both negative responses, such as sadness, distress, guilt, shock, and positive responses, such as compassion empathy, relief, gratitude, positive memories, hope and acceptance. Lessons learned focused primarily on patient care implications but included general life lessons. We also coded for institutional and informal support. In addition, our codebook incorporated concepts from loss and grief theories. Researchers coded essays for overall tone; intensity of emotions expressed; the emotional movement in the essay; and whether the essay overall focused on the loss experienced, either in negative or positive ways; or on implications for the future, whether negative or positive.

### Trustworthiness

Essays were coded by one investigator and quality controlled by another. Discrepancies were discussed until consensus was reached. We minimized bias through investigator triangulation (Denzin, 1978). Our team of researchers included members with diverse educational backgrounds and life experiences. These differences enabled us to approach the data from a variety of perspectives, compare divergent findings and reconcile interpretations. To monitor team reflexivity (Subramani, 2019), we kept personal notes regarding the project, and periodically shared insights. For example, one researcher’s experience of childhood loss made them especially sensitive to the way students regarded the suffering of family members. Another researcher’s experience of losing classmates/dealing with grief while in school contributed to their sensitivity regarding students’ lack of institutional support.

## Results

### Medical Student Characteristics

Sixty students participated in the study and 32 of the students (53.3%) were female. For privacy, all essays were blinded for any additional identifying student information prior to analysis.

### Student Narratives of Loss and Grief

Our analysis resulted in a conceptual model, “Medical Students’ Narratives of Loss and Grief” (Figure 1), showing the general pattern of student essays from the initial loss triggering negative emotions, followed by coping, positive emotions, and culminating in lessons learned. This model generally held true for essays of both personal and professional loss, while also indicating similarities and differences between the two.

**Figure 1.**
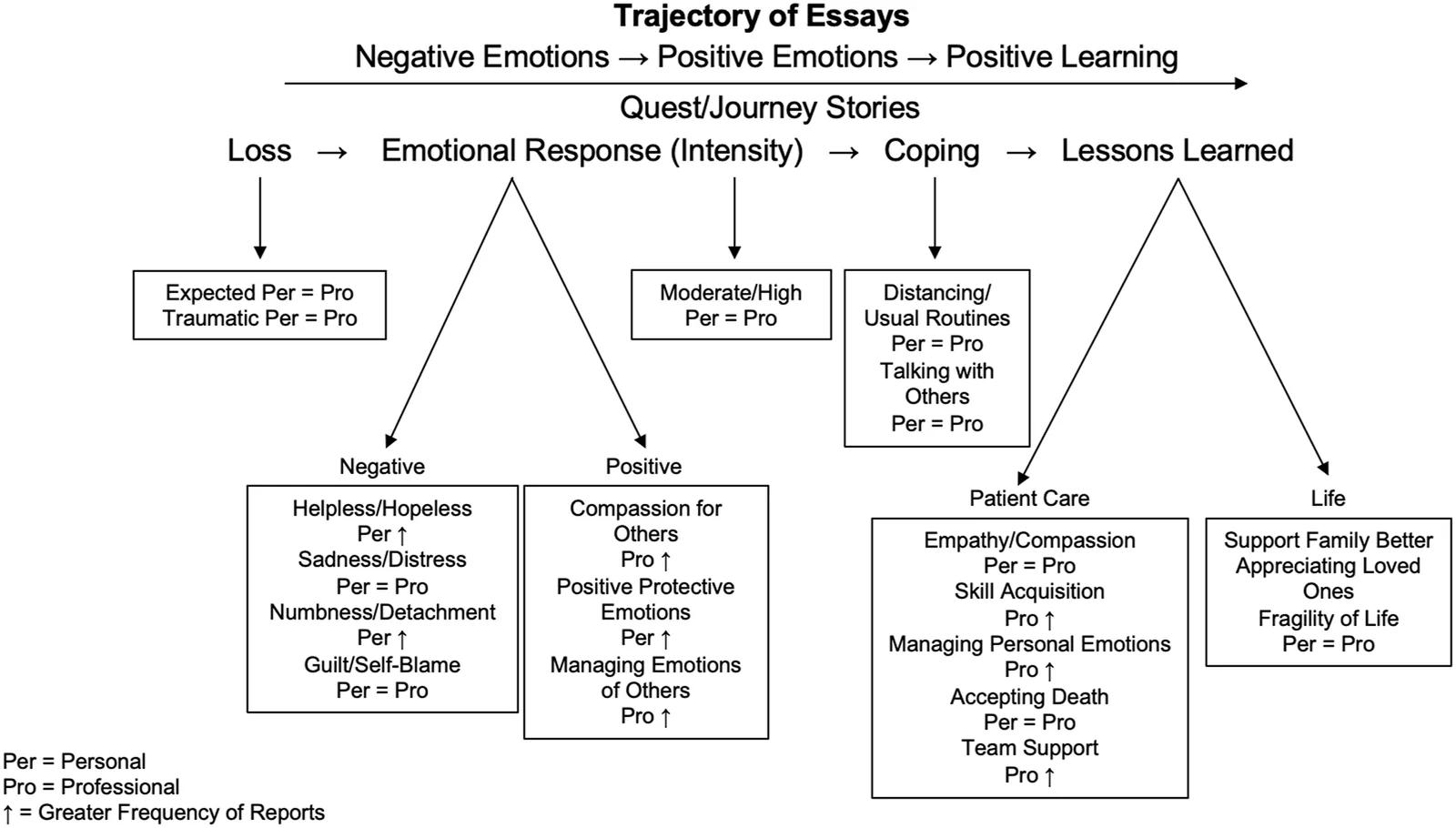
Medical students’ narratives of loss and grief.

#### Nature of Loss

More deaths were described as expected/peaceful than unexpected and traumatizing. In response to peaceful deaths, students tended to describe numb, detached feelings, but also compassionate feelings and positive reframing emotions. Traumatic deaths triggered similar negative and positive emotions, as well as additional negative emotions of sadness, guilt, anger, helplessness/hopelessness. Unexpected deaths seemed to evoke more complicated negative emotions in both groups, and similar compensatory psychological mechanisms when responding to an expected death.

Most students reported close or moderately close relationships with the deceased. Understandably, personal group students were closer to the deceased than professional group students. For the professional group, while their clinical responsibility for patients was more often low than high, their level of emotional involvement was more often high/moderately high, which may have accounted for their sense of closeness to these patients.

#### Students’ Emotional Reactions

The largest number of students reported compassion for patients or family:

“When I placed my hand on JA’s wife's shoulder to sympathize with and comfort her, I felt as though she was able to open up and explain her thoughts better to me. I was not simply a medical professional, but rather a kind ear to listen to her.” (1013PR)

This reaction was more common among professional group students. It was followed by various positive emotions and attitudes, which were more common for personal group students:

“I am also inclined to avoid painful topics and would rather keep all of my conversations cheery and positive.” (1001PE)

In almost equal numbers to positive responses, students reported feelings of helplessness and hopelessness:

“This was one of the only times I had seen him cry, and though it brought a heavy and almost gut-wrenching feeling to my gut, I couldn’t come up with anything remotely worthwhile to say.” (1001 PE)

Students also expressed emotions of sadness or distress:

“I’m not sure what I was expecting to happen, but I was hoping that we would have a moment of silence to respect the patient when it was all done and over with. But instead, Mr. M was left alone in his bed… It didn’t feel like a proper way for someone to pass, …” (1022PE)

Personal group students more frequently mentioned feeling helpless and hopeless, while sadness/distress occurred similarly in both groups.

Smaller numbers reported numbness/detachment. These students often coped by relying on routinized behavior and conscious distancing from the event.

“I couldn’t think of anything substantial to remember her by. I wondered why I didn’t cry. But I couldn’t stop and think – I had exams coming up, and I went back to my homework.” (1017PE)

Some students also experienced guilt/self-blame, more frequently for personal group students:

“Although the oncologist earnestly answered the family’s very specific questions about prognosis and treatment, I wonder if the family would have benefited more from discussions on their emotional state and acceptance of the patient’s disease.” (1029PR)

A small number of students described feeling angry.

A little under a quarter of all students focused on managing the emotions of others and this was almost entirely due to professional students:

“And as I held my 16-year old mother's hand, I let her know that I truly understood how she was feeling. A tear escaped from me, and the mother actually smiled.” (1003PR)

The main emotional responses to a patient or family member death were compassion for family, positive emotions/attitudes, helplessness and hopelessness, sadness and distress. Personal group students seemed to be more focused on their own grief, while professional group students focused more on the family of the deceased, especially in terms of expressing compassion toward them and helping them deal with their emotions. Personal group students were more likely to engage in positive emotions and cognitions about their loved one, perhaps out of a desire to rationalize the loss. Students in this group were also more likely to feel helpless and hopeless, perhaps because loss in a medical setting may be more expected than in a personal context.

#### Coping Strategies

Coping strategies were infrequently mentioned. Close to a third of students reported using strategies of detachment, distancing, and resorting to usual, routine behaviors:

“Surgeons are tough, I don’t want him to see my weakness. I wipe my tears and breathe deeply.” (1008PR).

Only about a fifth of the sample reported talking to others for support:

“I also remember how nice it was for my friends to show up for me.” (1005PE)

There were no differences in coping between the two groups.

Students appeared to be focused on describing their grief, rather than on how they managed it. Coping implies intentionality, so perhaps the idea of consciously managing grief, rather than just experiencing it, was not very much on their radar.

#### Lessons Learned

The large majority of students in both groups emphasized the importance of empathy and compassion:

“I think it is easy to cope by becoming numb to these situations when we are confronted with them over and over but I will strive to continue to feel and continue to try to aid the patient and their family in any way I can in those moments.” (1002PE)

The next most often mentioned lesson was the importance of skill acquisition, such as improved communication, which was higher for the professional group:

“Instead of sugar-coating or telling patients, “everything will be okay”, I have researched ways to speak to patients that shows them courtesy but does not give them false hope, while maintaining their dignity and our integrity as professionals.” (1014PR)

The importance of learning how to process and set boundaries on personal emotions was also important, more so for the professional than for the personal group. Smaller numbers mentioned accepting death and honoring patients’ wishes (about equal for both groups):

“This experience taught me the importance of respecting life – wherever a person is at in their life’s journey – the patient and family’s wishes...” (1016PR)

Additionally, the importance of team support was emphasized almost entirely in the professional group:

“But I felt like one of the most important lessons occurred while the team had a debriefing with every member of the ICU staff involved in her care. There I learned so many new aspects of difficulty providing care for severely sick patients,” (1002PR)

About a third of the sample for both groups stressed general life lessons, for example appreciating loved ones:

“Understanding the importance of losing my father’s parents has given me more appreciation for the time that I have to spend with my other grandparents.” (1016PE)

Students in personal and professional groups also recognized the fragility of life:

“For me, it was a great reminder of how short life can be and how important it is to cherish and appreciate every moment or interaction with people in our lives.” (1022PR)

Students in both groups emphasized the importance of empathy and compassion. About a third in each group noted general life lessons such as appreciating loved ones and valuing life. Skill acquisition and managing personal emotions were also frequently mentioned, mostly in the professional group.

#### Researchers’ Assessments

Researchers assessed global parameters based on their overall impressions of the essay which are depicted below in Figure 2.

**Figure 2.**
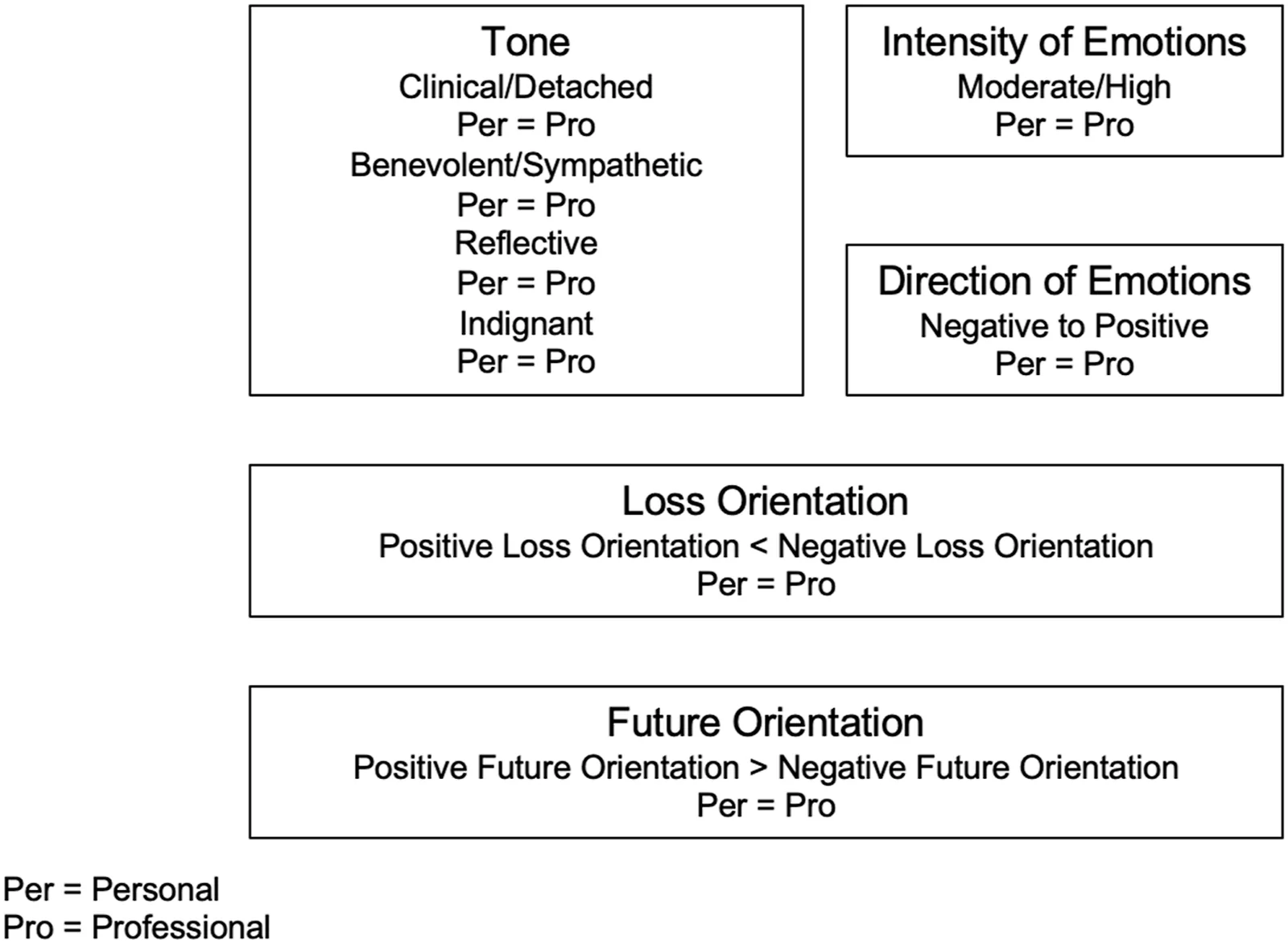
Overall assessment of students’ narratives of loss and grief.

According to these codes, tone was fairly evenly distributed across four categories of clinical/detached; benevolent/sympathetic; reflective; and indignant, regardless of type of loss. Emotional intensity of the writing tended to be high/moderately high in both groups.

The essays generally moved from negative to positive emotions for both personal and professional groups. A little less than half of the essays had an overall positive loss orientation, while a larger number had a negative loss orientation. Further, under half of essays expressed a positive future orientation, with almost no students reporting a negative future orientation. Again, both groups were similar.

## Discussion

The consistency of the data enabled us to identify a conceptual model describing medical student responses to loss and grief (Figure 1). While this model is an accurate reflection of our interpretation of the data, it revealed several surprising aspects.

First, although we expected experiences of personal and professional loss to be quite different from one another we found more similarities than differences. Personal group students did express more emotions, both positive and negative, but the intensity of the emotions was about equal between groups. One difference that was unexpected to us was professional group students’ greater emphasis on expressing compassion toward family members and managing the emotions of others. This finding might be explained by a tendency in this group to focus outward on dealing with patients’ families rather than attending to their own loss and grief.

Another unforeseen finding was how many positive responses such as compassion, gratitude, relief, positive memories students expressed. While the literature notes some of these responses, the preponderance of feelings reported is negative (Trivate et al., 2019). In our study, the number of negative and positive reactions was closer to equal.

A further unanticipated finding was that there was little mention of support in either group. Few essays noted family support, and virtually no essays reported institutional support, although some did describe compassionate attendings and supportive classmates. In recent years, medical schools have taken their responsibilities for student wellness more seriously, implementing a variety of support programs and initiatives (Álvarez-Montero et al., 2023), but on the whole, these essays presented a picture of students dealing with loss and grief in isolation. We were also startled to discover that there was fairly little reference to personal coping, although about a third mentioned distancing and taking refuge in routine behaviors, while a smaller number noted talking to others, both strategies mentioned in the literature (Dyrbye et al., 2019). Although many students did not discuss coping per se, it is possible to view the lessons learned as a form of cognitive/emotional coping. In this conceptualization, students tended to focus on useful, positive lessons that would restore a sense of being in control of these painful situations (Kelly & Nisker, 2010).

An additional finding we did not anticipate was the discrepancies between students and researchers’ perceptions. For example, although anger was rarely expressed overtly by students, about a quarter of the essays were judged to have an angry tone. Further, while students reported approximately equal frequencies for negative and positive emotions, as well as indicated a positive trajectory in their feelings, researchers evaluated essays as somewhat more likely to express a negative loss orientation than either a positive loss orientation or a positive future orientation.

In some respects, many aspects of our model fit within existing loss and grief frameworks. The kinds of emotions, both negative and positive, identified in both Kubler-Ross’ and Worden’s models, such as shock, numbness, anger, sadness, and acceptance, made their appearance in students’ essays (Corr, 2021; Worden, 2018). To a lesser extent, so did Worden’s tasks of mourning such as processing one’s emotions and resuming ordinary life. However, we did discover limitations of these models when applied to a cross-sectional understanding of medical students’ experiences. For example, we saw almost no evidence of the sort of oscillations described in the dual process model (Stroebe & Schut, 2010). Rather, although the rigid stage interpretation of Kubler-Ross’ model has been largely discredited, the essays seemed most likely to fit a fixed progression from difficult emotions to positive emotions and finally to constructive lessons learned.

Reasons for this lack of perfect fit likely have to do with the cross-sectional nature of our data set (although since students were reflecting on events that had occurred from several months to several years prior to the writing, they had ample opportunity to describe oscillations and nuance in their grieving process). We speculate that the predictable progression we found may also be related to the appeal of socially desirable narrative models. In other words, students may have felt most comfortable telling their stories in a simple, linear progression that reached a reassuringly upbeat resolution.

The large majority of essays in our study followed a narrative structure that most resembled a quest story (Frank, 2013). In storytelling typical of the quest narrative structure, these medical students saw themselves embarked on a journey to become physicians. Along the way, they encountered suffering and the pain of loss. Although they had almost no formal institutional support, they occasionally were able to identify individual wise guides and mentors who helped them deal with their losses. Ultimately, they learned valuable lessons that they could carry forward to make themselves better doctors.

While the relative uniformity of the essays may have been attributable in part to the phrasing of the assignment, the open-ended format and lack of grading suggest students could have responded in a variety of ways. We speculate that, as part of the informal curriculum (Lawrence et al., 2018), students may have concluded that the quest format is the preferred model for dealing with difficult events: acknowledge negative feelings, reframe the event more positively, and then figure out what they can learn from the experience to do better in the future. Although this homogenous approach may lead to resolution of difficult emotions, it does not allow space for messier, less predictable, more chaotic forms of response (Cohn et al., 2009).

This shared narrative structure may have resulted from a kind of collective narrative smoothing. Narrative smoothing is a research technique used to create coherence in narrative analysis by making choices about what to include, what to omit, where to focus, and what to emphasize (Coley et al., 2024). It is also something all people engage in when crafting a story. While an essential aspect of comprehensible narrative construction, excessive smoothing can result in homogenized stories that have lost some of their personal voice and authentic experience. In our study, narrative smoothing on the part of the student essayists may be one way of explaining our unexpected findings which is illustrated below in Figure 3.

**Figure 3.**
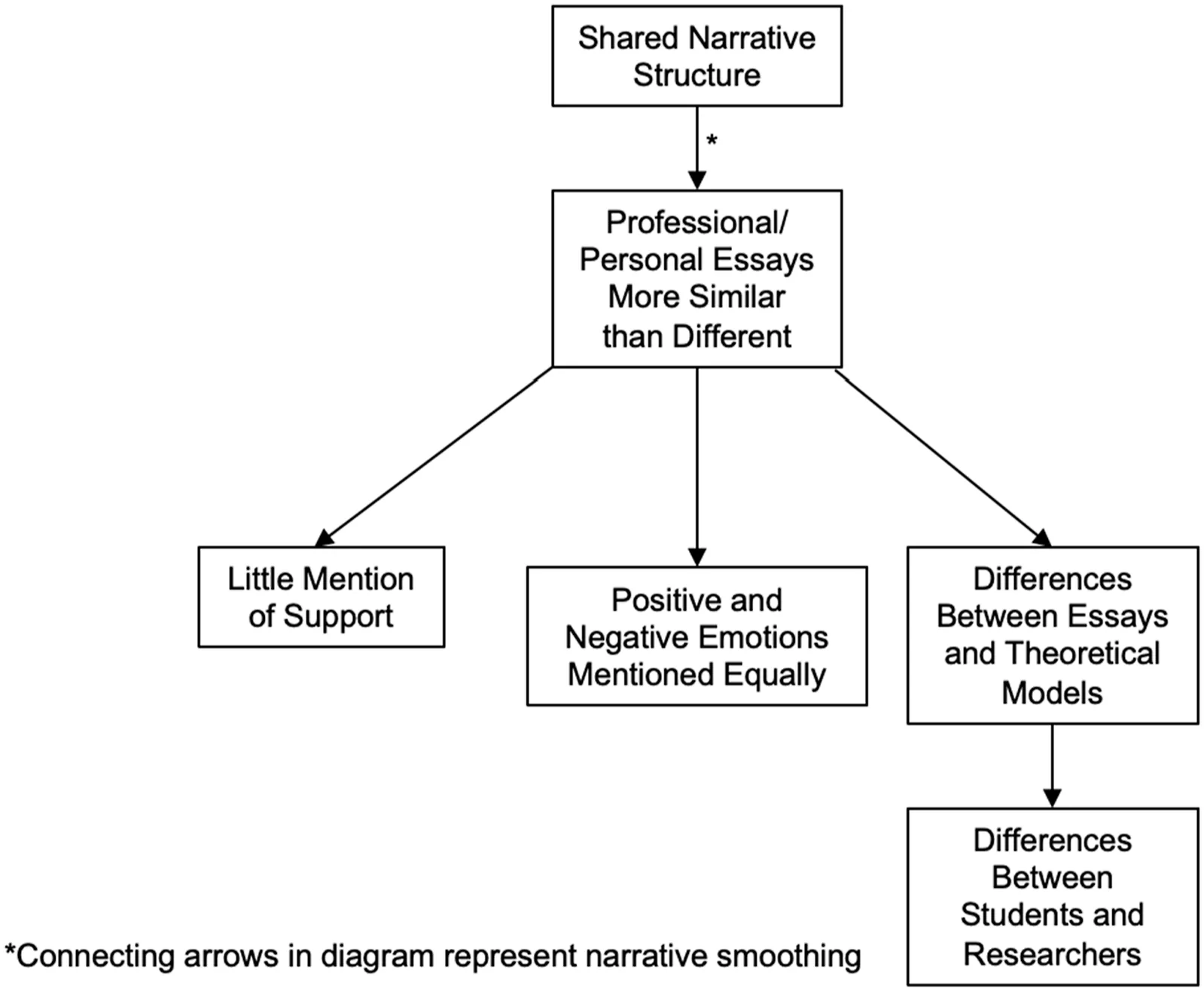
Narrative smoothing and shared narrative structure.

As students attempted, consciously or unconsciously, to homogenize their experiences to conform to a quest narrative, they inevitably converged toward the center. Student efforts to achieve a shared narrative structure may be a factor in why we discovered relatively few differences between personal and professional essays. The desire for narrative orthodoxy may also have contributed to students paying special attention to positive emotions and highlighting personal growth over institutional and familial support as a better fit with the quest storyline. Further, working within the constraints of this shared narrative structure could explain discrepancies between students’ and researchers’ interpretations of the essays as well as why differences emerged between the essays and theoretical models of loss and grief. More research is needed to investigate these relationships further.

We used high quality data to examine variation in medical student experiences of personal and professional loss. However, several aspects of the data and study limit our conclusions. First, while the goal of qualitative research is not generalizability, because the study was monocentric in the context of a single public medical school, this may have skewed the findings, though theoretical saturation was achieved. Second, the data could be impacted by self-enhancement bias (Shepperd et al., 2008) and other memory biases (Hertel & Matthews, 2011Hertel & Mathews, 2011) since the data consisted of students’ retrospective recollections of events. Greater reporting accuracy is suggested by the level of detail in these recollections. Larger, quantitative studies can make meaningful contributions about generalizability of results to students at other institutions.

## Conclusion

Medical students in this study reported similar reactions to personal and professional loss, with some expected differences, such as students who experienced professional loss more often noting compassion for others and more frequently discussing managing the feelings of others, the importance of skill acquisition, processing personal emotions and team support. Students experiencing personal loss understandably appeared more focused on their own grief and more often commented about feelings of helplessness and numbness. Students in both groups reported little about how they coped with their grief or about receiving either informal or institutional support. The similarity of the essays, while due to many factors, may suggest internalizing pressures to conform to socially desirable narratives. Medical educators and clinical supervisors should help students develop effective coping skills in response to loss, provide better institutional support, and encourage students to tell authentic stories about their experiences of loss and grief.
